# Multiple Sclerosis Patient Management During the COVID-19 Pandemic: Practical Recommendations From the Portuguese Multiple Sclerosis Study Group (GEEM)

**DOI:** 10.3389/fneur.2021.613769

**Published:** 2021-03-08

**Authors:** João J. Cerqueira, Ana F. Ladeira, Ana M. Silva, Ângela Timóteo, José Vale, Lívia Sousa, Marta Arenga, Pedro Abreu, Rui Guerreiro, João de Sá

**Affiliations:** ^1^Life and Health Sciences Research Institute (Instituto de Investigação em Ciências da Vida e Saúde), School of Medicine, University of Minho, Braga, Portugal; ^2^Neurology Department, Centro Hospitalar Universitário Lisboa Central, Lisbon, Portugal; ^3^Department of Neurology, Centro Hospitalar e Universitário Do Porto, Porto, Portugal; ^4^Department of Neurology, Hospital Beatriz Ângelo, Loures, Portugal; ^5^Department of Neurology, Centro Hospitalar e Universitário de Coimbra, Coimbra, Portugal; ^6^Department of Neurology, Centro Hospitalar da Cova da Beira, Lisbon, Portugal; ^7^Department of Neurology, Centro Hospitalar São João, Porto, Portugal; ^8^Department of Neurology, Centro Hospitalar de Setúbal, Setúbal, Portugal; ^9^Department of Neurology, Centro Hospitalar Lisboa Norte, Lisbon, Portugal

**Keywords:** telehealth, virtual consultation, monitoring, remote assessment, consensus

## Abstract

The spread of the COVID-19 pandemic has imposed significant challenges on healthcare provision, requiring changes in the conventional patient management, particularly in chronic diseases like multiple sclerosis (MS). To increase patient safety and reduce the risk of infection, while ensuring an appropriate and regular follow-up, tele-medicine gained prominence as a valid alternative to face-to-face appointments. However, the urgency of the implementation and the lack of experience in most MS centers led to “*ad hoc*” and extremely diverse approaches, which now merit to be standardized and refined. Indeed, while tele-consultation cannot fully replace face-to-face visits, it certainly can, and will, be incorporated as part of the routine care of MS patients in the near future. Bearing this in mind, the Portuguese Multiple Sclerosis Study Group (GEEM) has developed a set of recommendations for the usage of tele-medicine in the management of MS patients, both during the pandemic and in the future. The consensus was obtained through a two-step modified Delphi methodology, resulting in 15 recommendations, which are detailed in the manuscript.

## Introduction

Multiple sclerosis (MS) is a chronic degenerative disorder of the central nervous system of auto-immune origin (1) that affects 2.8 million persons worldwide and 0.142% of Europeans (2). Optimal management of MS includes early diagnosis and treatment as well as regular monitoring and follow-up, since patients often need their treatments to be adjusted for lack of efficacy, tolerability, or safety issues (3). In general, patients with MS are followed periodically in specialized MS clinics (approximately every 6 months) with visits that include, as a minimum, a clinical interview for any new symptoms, adverse events and relapses, a neurological examination, and a review and discussion of any ancillary examinations performed (4). In addition, given their importance for the optimal control of the disease, patient education regarding diet, physical exercise, smoking, and other lifestyle factors should also take place at each visit (4).

The infection by the new respiratory agent SARS-CoV-2 was firstly identified in China in December 2019 (5). On March 11, 2020, the World Health Organization declared COVID-19 (as the disease caused by SARS-CoV-2 was named) a pandemic (6). In Portugal, the first case was registered on March 2, and 14 days later, the Ministry of Health determined that all non-urgent care provision should be canceled, redirecting all efforts to fight COVID-19 (7). This suspension was lifted at the beginning of May (8), but by then, thousands of appointments and exams had been canceled and had to be rescheduled. Moreover, mandatory safety measures, such as limiting the presence of patients in the waiting rooms and introducing “empty periods” in the schedule to accommodate delays in face-to-face visits, significantly limited the availability of healthcare services. This was further complicated by the fact that many MS patients avoided going to healthcare facilities, for fear of acquiring SARS-CoV-2 (9).

From the beginning of the pandemic, tele-medicine has emerged in several countries as a possible solution for balancing the need to prevent infection with the need to keep an appropriate follow-up (10–20). Tele-medicine enables the regular and close contact between patient and physician while lowering the need for patient's physical presence in Health Units, and hence the risk of exposure to SARS-CoV-2 (14, 20). The implementation of tele-medicine, in particular tele-consultations, should not rely on *ad hoc* approaches by individual physicians, but rather be based in internationally recognized best practices and, as much as possible, standardized (14). In the last year, many publications addressed the care of MS under a global pandemic, describing cases of success (16, 17, 19), giving practical recommendations (11–14), and sometimes focusing on particular aspects of the teleconsultation (15). However, clear, systematic, consensus-based recommendations that reflect the opinion of diverse group of practitioners and can be widely adopted are largely missing, with two notable exceptions coming from Latin America (18, 20) and Italy (21). Moreover, while attention has been paid to the content of the tele-consultation (18, 20, 21), there is a gap regarding the entire tele-consultation organization and management. Recognizing this gap, the Portuguese Multiple Sclerosis Study Group has developed a document with recommendations for using tele-consultation/telemedicine to manage MS patients. This document is a collection of best practices developed by neurologists with strong expertise in MS and was developed bearing in mind that tele-medicine will inevitably be, to a greater or lesser extent, incorporated in the routine care of MS patients in the near future.

Although this is not a formal and exhaustive guideline document, it is expected that this expert consensus may provide some guidance to physicians in the best approach to use tele-medicine in MS patients.

## Methods

The consensus was obtained through a two-step modified Delphi methodology that took place between June and July 2020 and consisted of one round of online questionnaire followed by a virtual consensus meeting.

A comprehensive list of items for evaluation was initially developed, aligned with the five fundamental steps in patient management: triage, appointment, follow-up, nursing, and communication (as presented in Table 1).

**Table 1:** Organization of topics for the development of recommendations.

**Section**	**Addressed topics**
Triage	Relevance of tele-screening Data collection
Appointment	Importance of tele-medicine Eligibility criteria for non-face-to-face appointment Barriers for tele-medicine
Follow-up	Patient monitoring changes Disease dashboards Self-reporting monitoring tools Relapse procedure
Nursing	Nursing role Nurse non-face-to-face appointment
Communication	Communication channels Information sharing

The questionnaire was developed aligned with this topic list, considering both open-ended questions (like “*which are the main benefits of tele-medicine?”*) and closed-ended questions (such as “*of the following, which are the relevant criteria for tele-appointment eligibility?”*). The questionnaire is presented as Supplementary Material.

A total of 158 neurologists were invited for the online questionnaire round. Thirty different responses were obtained, from respondents that had, on average, 15 years of experience in MS management. These physicians followed at least 150 patients and were evenly split among the three main health regional administrations (North, Center, and “Lisboa and Vale do Tejo” —LVT), with additional colleagues from the autonomous regions of Azores (1) and Madeira (2). The latter were invited to increase the generalizability of the recommendations. While the provision of care to persons with MS in Madeira and Azores is broadly similar to that of mainland Portugal, the two regions have their own regional health services with corresponding specificities that should be taken into consideration.

Questionnaire results were translated into a report with a preliminary set of recommendations. Resulting statements progressed to the second round of Delphi consensus regardless of the agreement level, but with the indication of the percentage of agreement between experts, to be further considered. These were discussed in a virtual consensus meeting with eight experts, and final recommendations were developed and sent for validation.

The Delphi panel was considered an appropriate methodology for obtaining practical guidelines on MS patients' management as it ensures anonymity between participants, iteration with controlled feedback of group opinion, statistical aggregation of group response, and expert input (22).

## Section 1: Triage

Triage is a very important and often disregarded activity. An effective triage may highly increase the effectiveness of the appointment, enabling an adequate preparation while saving physicians' time (13).

Participants were asked about distinct procedures to optimize the triage process, mainly related to tele-triage and patient data collection prior to the specialist appointment.

### Actionable Recommendations

1. The implementation of a system to collect patient information before the appointment is highly recommended. Data collection should be performed through a tele-screening process with the specialized nurse, or a specific and certified tool, accessible to both patients and healthcare professionals.
  The collected data should include new symptoms, relapses, exams results, ability to perform daily tasks, and current therapy.

## Section 2: Appointment

Health systems are trying to adapt themselves to this new reality: creating distinct circuits for COVID-19 and non-COVID-19 patients and adjusting both facilities and procedures to the safety recommendations. This adaption has caused some disruption in the regular healthcare provision, delaying appointments, exams, and procedures. Although tele-medicine (and particularly phone contacts) has been widely used to mitigate the limitation in physical availability, there is a need to define an appropriate framework for non-face-to-face appointment, ensuring its effectiveness (14).

Most of the Delphi questionnaire was related to the appointment. Participants were asked to evaluate the importance of tele-medicine and its suitability to the MS context. From a list of possible criteria for non-face-to-face appointments (that included disability status, relapses, therapy, geographic location, risk profile, and age, among others), physicians rated the importance of each variable and defined the necessary conditions, reaching a consensus on the eligibility for a non-face-to-face appointment. As non-face-to-face appointment effectiveness is often limited by both infrastructural and personal constraints, participants discussed the required steps to ensure tele-medicine adoption.

### Actionable Recommendations

2. Non-face-to-face appointment (tele-appointment or video-appointment) is a good alternative to face-to-face appointment, as it reduces the risk of exposure to the virus, facilitates physician access, and could be more convenient to patients and caregivers, preventing a hospital visit. However, non-face-to-face appointments should only be an alternative during a limited time period, since physicians still need to physically examine MS patients on a regular basis.
3. The ideal follow-up frequency for previously diagnosed and stable patients should be every 6 months, with at least one face-to-face appointment per year.
4. The following criteria should be met to determine the eligibility for a non-face-to-face appointment:
  - Diagnosed patients, coming for follow-up and treatment monitoring; non-face-to-face appointments should not be used to establish or discuss a new MS diagnosis
  - Stable patients, without current or recent relapse suspicion; patients with a suspected or recent relapse should be examined in person
    In addition, the patients living far from the hospital, or with accessibility constraints, and patients with high disability level are most likely to benefit from non-face-to-face appointments.
5. During non-face-to-face appointments, as in face-to-face appointments, a set of relevant information should be evaluated and recorded in the patient registry:
  - New symptoms and relapses;
  - Treatment adverse effects/changes to the current therapy;
  - Other comorbidities;
  - Urinary/intestinal complaints;
  - Ability to walk;
  - Cognitive complaints;
  - Remote neurological examination (in case of video-appointment).

Besides these vital data, other aspects should also be evaluated, such as fever/infection, fatigue, depression, risk of social isolation, plans to start a family, and information about labor activity.

6. Video-appointment is preferable to tele-appointment, as it enables a stronger and closer connection between patient and physician. Moreover, visual evaluation provides additional clinical information.
7. To reduce the difficulties and resistance that are often associated with digital and tele-medicine, ensuring healthcare professionals' adoption to digital appointments, hospitals should:
  - Provide adequate means for video-appointments: setup videoconferencing platforms (Teams, Zoom), acquire the necessary devices (cameras, computers with adequate capacity, appropriate internet connection), and make platforms for document sharing available;
  - Train the clinical team;
  - Ensure the prior patient preparation for this type of appointment.
8. Patient acceptance of tele-appointments should be ensured by:
  - Regularly updating patients' contact information;
  - Raising awareness for the effectiveness of non-face-to-face appointments (this should be performed by all relevant stakeholders: clinical team, nursing team, hospital, patients' association and the Portuguese MS Study group);
  - If necessary, requiring the presence of a caregiver on non-face-to-face appointments to support patients (to hold the phone during coordination exercises, record walking exercise, etc.), particularly when there is a high disability level.

## Section 3: Follow-Up

Besides follow-up appointments, MS patients require regular monitoring to evaluate relapses and disease progression (4). The limitation in the availability of ancillary exams induced by the pandemic has created some variability in the way different physicians and hospitals manage their patients.

Participants discussed monitoring frequency and procedures and required exams for an adequate MS patient management. Additional tele-monitoring tools were also evaluated and deemed relevant, although there was no consensus on the most appropriate tool.

### Actionable Recommendations

9. Due to restrictions in healthcare facilities, MS monitoring protocol may be adjusted. It is recommended to:
  - Postpone exams that are not related to the disease safety protocol;
  - Increase the interval of routine exams, such as blood and MRI tests in stable patients. However, the safety protocol exams are still vital for patients' follow-up, regardless of the pandemic context. Similarly, in urgent situations, all procedures deemed necessary should not be postponed.

In addition, patients should send the exams results (performed outside the hospital) to the institutional e-mail of the attending physician, avoiding an unnecessary hospital visit.

10. As a follow-up complement, online questionnaires such as PDDS (*Patient Determined Disease Steps*), MSQol-54 (*MS Quality of Life*), MSIS-29 (*MS Impact Scale*), MSWS-12 (*MS Walking Scale*), and other monitorization tools (such as apps for cognitive evaluation and wearables) may also be used.
11. If there is a suspected relapse, the procedure should be the same as before the pandemic:
  - Tele-screening should be used to evaluate the symptoms and clinically validate the relapse;
  - Corticosteroids should still be administered, if there is clinical justification;
  - Intravenous administration should be maintained, as in the pre-pandemics context. However, for certain mild relapses, oral corticosteroids can be prescribed, reducing patient visits to the hospital.
  - These situations should be individually analyzed considering the patient profile. The decision to request the patient presence in the hospital due to a suspicion/occurrence of a relapse or the decision to continue the corticosteroid therapy should be taken by the physician balancing the benefits and the risks of each option.

## Section 4: Nursing

Nursing care is crucial to adequate patient management, both in a face-to-face and in a non-face-to-face context. In the latter, a nurse role may be even more relevant, as counseling and education are vital to ensure that patients can adequately manage their disease in their homes.

Participants discussed the necessary coordination between physicians and nurses and the relevance of specialized staff.

### Actionable Recommendations

12. Nurses also play a fundamental role in non-face-to-face appointments, due to the relevance of counseling and education. A useful and effective non-face-to-face nursing appointment should:
  - Be scheduled in the interval of physicians' appointments;
  - Occur as a video-appointment, focused on the patients' education.
13. The hospital should ensure the presence of nursing staff specialized in MS. Nurses' training is fundamental, and an appropriate task division (considering roles, data to collect, and disease scales to assess) should be performed, ensuring there is no overlap and duplication between physicians and nursing appointments.

## Section 5: Communication

Communication is key in healthcare provision—an effective communication promotes patients' knowledge, reassures patients and caregivers, fosters patients' compliance with the treatment plan and can immediately increase patient perception on the quality of care provided (4).

The communication with the patient is even more important in this pandemic context—patients must be informed of the necessary care procedures and treatment continuity, so they feel confident in their disease management even if they are not able to go to the hospital.

Participants were asked to evaluate the importance of different communication channels, discussing the type of information to be shared with patients to ensure appropriate communication flow.

### Actionable Recommendations

14. It is critical to reinforce an effective transmission of information to the patient by:
  - Sharing relevant information with patients by e-mail or message;
  - Communicating relevant information through Patients' Associations and the Portuguese MS Study Group—promotion of a healthy lifestyle and reliable information update;
  - Creating a support line for patients with MS.
15. It is important that physicians are truly available to answer patients' doubts when they have a chronic disease like MS. Patients' direct contact with their care team (healthcare professionals that follow them in a regular basis) should be privileged.

## Discussion

The set of recommendations reflects the experience of relevant physicians in MS management in Portugal. While the scientific investigation on COVID-19 is moving forward and there is low evidence regarding the appropriate patient management in the pandemic context, the follow-up of MS patients should be based on the above-mentioned recommendations.

These recommendations are meant to reduce patient risk of contagion, by avoiding unnecessary hospital visits and fostering the usage of tele-medicine, while ensuring a standardization of MS patient management. These measures also consider this context of reduced healthcare services availability.

Some of these measures are easy to implement, while others require infrastructure changes or investments. Additionally, clinical judgement is paramount, and these recommendations should only be applied to patients that meet the defined criteria and when the usage of digital channels will not reduce the effectiveness of patient follow-up. These recommendations should be enforced in alignment with patient-specific factors and hospital procedures.

With the advance of research on both COVID-19 and the impact of this disease in MS patients, it is expected that further updates and more substantive guidelines can be developed.

## Data Availability Statement

The original contributions presented in the study are included in the article/Supplementary Material, further inquiries can be directed to the corresponding author/s.

## Author Contributions

JJC and JdS had the initial idea and developed the work plan. JJC coordinated the project. JJC and AFL wrote the first draft of the paper. JJC, AFL, AMS, AT, JV, LS, MA, PA, RG, and JdS participated in the consensus meeting, where the final recommendations were produced, and contributed to the manuscript. All authors reviewed and approved the manuscript.

## Conflict of Interest

The authors declare that the research was conducted in the absence of any commercial or financial relationships that could be construed as a potential conflict of interest.
