# Supplementary material for: Multiple Sclerosis Patient Management During the COVID-19 Pandemic: Practical Recommendations From the Portuguese Multiple Sclerosis Study Group (GEEM)
Source: Front Neurol. 2021 Mar 8;12:613769. doi: 10.3389/fneur.2021.613769 (PMC8006454; doi:10.3389/fneur.2021.613769)
Supplement: Supplementary file 1 [file Data_Sheet_1.docx]

**Questionário: Seguimento dos Doentes com Esclerose Múltipla durante a COVID-19**

A telemedicina tem sido apresentada como uma possível solução que permite que os doentes evitem deslocar-se a unidades de saúde para a realização de consultas, de maneira a diminuir o risco de exposição ao novo coronavírus SARS-CoV-2.

No caso dos doentes com esclerose múltipla, este acompanhamento virtual pode ter de ser conjugado com visitas presenciais aos cuidados de saúde em virtude da necessidade de realização de exames ou de tratamentos intravenosos.

**Q1:** A teletriagem pode ser implementada como forma de analisar a história clínica recente do doente previamente à consulta. Que informações considera que devem ser pedidas aos doentes nesta fase, de modo a definir os seus níveis de prioridade e avaliar a necessidade de consulta? *[resposta aberta]*

**Q2:** Considera a realização de teleconsultas não presenciais uma possível alternativa às consultas presenciais? Quais os principais benefícios deste tipo de atividade? *[resposta aberta]*

**Q3:** Que critérios considera relevantes para a seleção da tipologia de consulta (presencial ou não presencial)? *[incluir uma lista de critérios e deixar a opção “Outras” em aberto]*

- Consulta de diagnóstico *vs* consulta de seguimento;
- Idade dos doentes;
- Perfil de risco (outras comorbilidades);
- Localização geográfica;
- Grau de incapacidade;
- Terapêutica específica;
- Análises que deva realizar;
- Efeitos adversos;
- Falência terapêutica;
- Suspeita de surto;
- Outras (quais?)

**Q4:** Tendo em conta os critérios que definiu anteriormente como relevantes, que perfil de doentes selecionaria para consulta não presencial?

- Consulta de diagnóstico vs consulta de seguimento *[resposta aberta]*
- Idade dos doentes *[resposta aberta]*
- Perfil de risco (outras comorbilidades) *[resposta aberta]*
- Localização geográfica *[resposta aberta]*
- Grau de incapacidade *[resposta aberta]*
- Terapêutica específica *[resposta aberta]*
- Análises que deva realizar *[resposta aberta]*
- Efeitos adversos *[resposta aberta]*
- Falência terapêutica *[resposta aberta]*

**Q5:** A consulta não presencial pode ser realizada pelo telefone ou através de plataformas de videoconferência. Qual a alternativa que considera mais importante para uma consulta com um doente de Esclerose Múltipla? Fundamente a sua resposta. Qual a alternativa que utiliza atualmente? *[resposta aberta]*

**Q6:** Que dificuldades antevê na realização de consulta não presencial? Como acredita que estas dificuldades poderiam ser ultrapassadas? *[resposta aberta]*

**Q7:** Gostaria de ter informação enviada pelo doente antes da consulta?

*[resposta aberta]*

**Q8:**Que tipo de informação pode ser enviada pelo doente antes da consulta de maneira a facilitar a avaliação do mesmo durante a consulta? *[resposta aberta]*

**Q9:** Qual o conjunto mínimo de dados que devem ser recolhidos nesta consulta não presencial? *[resposta aberta]*

**Q10:** Considera necessária a ajuda de um cuidador ou pessoa de referência para a realização de exames e avaliações através de vídeo-consulta? A sua resposta depende do perfil do doente, no que diz respeito ao seu grau de incapacidade físico e/ou cognitivo? *[resposta aberta]*

**Q11:** Que exames, análises de seguimento e avaliação funcional podem ser mantidos no regime de consulta não presencial (quais são possíveis de realizar através da teleconsulta ou da vídeo-consulta)? *[resposta aberta]*

**Q12:** Por outro lado, que exames e análises podem deixar de ser realizados / não são fundamentais para a avaliação da evolução do doente numa consulta não presencial? *[resposta aberta]*

**Q13:** Durante a pandemia pondera alterar o numero de análises / exames / avaliações pedidas ou cuidados assistenciais face ao período anterior ao COVID-19? Porquê? *[resposta aberta]*

**Q14:** Considera que o envio por email dos resultados dos exames realizados é uma boa alternativa à deslocação ao hospital por parte do doente? Fundamente a sua resposta *[resposta aberta]*

**Q15:** Acredita que a implementação de questionários online como ferramenta de monitorização dos doentes é uma ferramenta útil para o seu seguimento? Fundamente a sua resposta *[resposta aberta]*

**Q16:** Que outras ferramentas de telemonitorização sugere? *[resposta aberta]*

**Q17:** Em alguns hospitais podem não existir condições para a realização da videoconsulta, podendo ser necessário optar pela teleconsulta. Que informações relativamente à evolução do tratamento e da doença consegue obter à luz desta alternativa, de maneira a produzir uma avaliação precisa? *[resposta aberta]*

**Q18:** Qual é a frequência ideal de acompanhamento destes doentes? Qual a combinação ideal entre consulta presencial e não presencial? *[resposta aberta]*

**Q19:** Como vê o papel da enfermagem nas consultas não presenciais? A sua relevância é diferente nas consultas não presenciais do que nas consultas presenciais? Fundamente a sua resposta *[resposta aberta]*

**Q20:** Qual considera ser o procedimento adequado na ocorrência de um surto? Deverá ser mantida a administração de corticosteróides durante a pandemia? Em caso afirmativo, considera que estes possam ser administrados de modo oral em vez de intravenoso nesta altura? *[resposta aberta]*

**Q21:** Quais as suas recomendações relativamente aos autocuidados que o doente deve ter durante este período de pandemia? *[resposta aberta]*

**Q22:** Que medidas podem ser tomadas para manter os doentes informados relativamente aos cuidados que devem ter durante a pandemia e como devem proceder relativamente à continuidade dos tratamentos? *[resposta múltipla]*

- Criar um fórum online com doentes, médicos e enfermeiros, que possibilite a partilha de informação e permita aos doentes colocar questões;
- Partilhar informação relevante com os doentes através do email ou SMS;
- Transmitir a informação necessária aos doentes aquando da consulta ou teleconsulta;
- Criar uma linha de apoio aos doentes;
- Outra.

Seleccione todas as que considera relevante, fundamentando a sua resposta. *[resposta aberta]*
